# A Bayesian network model for prediction of low or failed fertilization in assisted reproductive technology based on a large clinical real-world data

**DOI:** 10.1186/s12958-023-01065-x

**Published:** 2023-01-26

**Authors:** Tian Tian, Fei Kong, Rui Yang, Xiaoyu Long, Lixue Chen, Ming Li, Qin Li, Yongxiu Hao, Yangbo He, Yunjun Zhang, Rong Li, Yuanyuan Wang, Jie Qiao

**Affiliations:** 1 Center for Reproductive Medicine, Department of Obstetrics and Gynecology, Peking University Third Hospital, Beijing, China; 2 National Clinical Research Center for Obstetrics and Gynecology (Peking University, Third Hospital), Beijing, China; 3 Key Laboratory of Assisted Reproduction (Peking University), Ministry of Education, Beijing, China; 4 Beijing Key Laboratory of Reproductive Endocrinology and Assisted Reproductive Technology (Peking University Third Hospital), Beijing, China; 5 School of Mathematical Sciences, LMAM, LMEQF, and Center of Statistical Science, Peking University, Beijing, China; 6 School of Public Health, Peking University, Beijing, China; 7 Beijing Advanced Innovation Center for Genomics, Beijing, China; 8 Peking-Tsinghua Center for Life Sciences, Peking University, Beijing, China

**Keywords:** In vitro fertilization, Intracytoplasmic sperm injection, Fertilization failure, Bayesian network, Prediction model

## Abstract

**Study question:**

To construct prediction models based on the Bayesian network (BN) learning method for the probability of fertilization failure (including low fertilization rate [LRF] and total fertilization failure [TFF]) in assisted reproductive technology (ART) treatment.

**Summary answer:**

A BN model was developed to predict TFF/LFR. The model showed relatively high calibration in external validation, which could facilitate the identification of risk factors for fertilization disorders and improve the efficiency of in vitro fertilization/intracytoplasmic sperm injection (IVF/ICSI) treatment.

**What is known already:**

The prediction of TFF/LFR is very complex. Although some studies attempted to construct prediction models for TFF/LRF, most of the reported models were based on limited variables and traditional regression-based models, which are unsuitable for analyzing real-world clinical data. Therefore, none of the reported models have been widely used in routine clinical practice. To date, BN modeling analysis is a prominent and increasingly popular machine learning method that is powerful in dealing with dynamic and complex real-world data.

**Study design, size, duration:**

A retrospective study was performed with 106,640 fresh embryo IVF/ICSI cycles from 2009 to 2019 in one of China's largest reproductive health centers.

**Participants/materials, setting, methods:**

A total of 106, 640 cycles were included in this study, including 97,102 controls, 4,339 LFR cases, and 5,199 TFF cases. Twenty-four predictors were initially included, including 13 female-related variables, five male-related variables, and six variables related to IVF/ICSI treatment. BN modeling analysis with tenfold cross-validation was performed to construct the predictive model for TFF/LFR. The receiver operating characteristic (ROC) curves and the corresponding area under the curves (AUCs) were used to evaluate the performance of the BN model.

**Main results and the role of chance:**

All twenty-four predictors were first organized into seven hierarchical layers in a theoretical BN model, according to prior knowledge from previous literature and clinical practice. A machine-learning BN model was generated based on real-world clinical data, containing a total of eighteen predictors, of which the infertility type, ART method, and number of retrieved oocytes directly influence the probabilities of LFR/TFF. The prediction accuracy of the BN model was 91.7%. The AUC of the TFF versus control groups was 0.779 (95% CI: 0.766-0.791), with a sensitivity of 71.2% and specificity of 70.1%; the AUC of of TFF versus LFR groups was 0.807 (95% CI: 0.790-0.824), with a sensitivity of 49.0% and specificity of 99.0%.

**Limitations, reason for caution:**

First, our study was based on clinical data from a single center, and the results of this study should be further verified by external data. In addition, some critical data (e.g., the detailed IVF laboratory parameters of the sperm and oocytes used for insemination) were not available in this study, which should be given full consideration when further improving the performance of the BN model.

**Wider implications of the findings:**

Based on extensive clinical real-world data, we developed a BN model to predict the probabilities of fertilization failures in ART, which provides new clues for clinical decision-making support for clinicians in formulating personalized treatment plans and further improving ART treatment outcomes.

**Study funding/competing interest(s):**

Dr. Y. Wang was supported by grants from the Beijing Municipal Science & Technology Commission (Z191100006619086). We declare that there are no conflicts of interest.

**Trial registration number:**

N/A.

**Supplementary Information:**

The online version contains supplementary material available at 10.1186/s12958-023-01065-x.

## Introduction

Fertilization is the most crucial step during in vitro fertilization (IVF) and intracytoplasmic sperm injection (ICSI) in the field of assisted reproductive technology (ART). Fertilization results from complex processes and reactions between sperm and oocytes [1, 2]. Any breakdown in this process could lead to fertilization disorders, including a low fertilization rate (LFR, defined as a fertilization rate lower than 25%) or even total fertilization failure (TFF, defined as no fertilized egg formation and a fertilization rate of zero) [3]. The occurrence of an LFR ranges from 10 to 20% in IVF, while that of TFF is 10%-20% in IVF and even 3–5% in ICSI [4, 5]. The TFF/LFR could result in the failure of IVF/ICSI treatment, thus placing heavy economic and psychological burdens on couples undergoing IVF/ICSI cycles.

The prediction of TFF/LFR is very complex. Although the laboratory parameters of retrieved sperm and oocytes have been proposed to be essential factors in the fertilization rate [6, 7], it has been reported that TFF can also occur in couples with apparently normal gametes with good quality [8], implying that there might be other important factors that affect fertilization. Recently, many studies have reported that disrupted genetic and epigenetic patterns [9, 10], female age and duration of infertility [11], and female hormone levels [12, 13] could contribute to the risk of fertilization failure.

Some studies have attempted to develop prediction models for the occurrence of fertilization disorders based on traditional regression models. One study included 304 couples with TFF and 304 control couples to develop a prediction model for TFF [14], while another study included 892 couples to develop a TFF prediction model [15]. These reported models are based on minimal sample sizes and variables, and none have been widely used in routine clinical practice [16]. In our previous study, we used an extensive clinical database based on IVF medical records and established a prediction model for TFF/LFR using multiple logistic regression models [17]. Traditional regression-based approaches have the following features: 1) they model associations rather than causal structures; 2) they operate under restrictive assumptions about the relationships among variables, and the relationships between predictors cannot be considered; and 3) each outcome must be trained on the model and a static set of data [18]. These features make the roles of conventional regression-based prediction models quite limited, especially for dynamic and complicated clinical data.

Machine learning algorithms for constructing personalized risk prediction models have been extensively developed. The Bayesian network (BN) is one of the most widely used machine learning approaches for risk prediction [19]. The structure of a BN model is a directed acyclic graph (DAG), where the arcs have a formal interpretation in terms of probabilistic conditional independence [20]. BN models are powerful for investigating dependent relationships among the variables of a domain under uncertainty and dealing with real-world data that are dynamic and complex [19].

Therefore, we performed a retrospective study based on a real-world clinical database that involved 106,640 IVF/ICSI cycles from 2009 to 2019 in a reproductive center. In this study, we aimed to develop a novel BN model with better performance. The model accurately and dynamically predicted the possibilities of TFF/LFR by using known predictors obtained from clinical practice. This study could provide new clues for clinical decision-making support for clinicians in formulating personalized treatment plans and further improving the outcomes of IVF/ICSI treatment.

## Materials and methods

### Study population

**Fig. 1:**
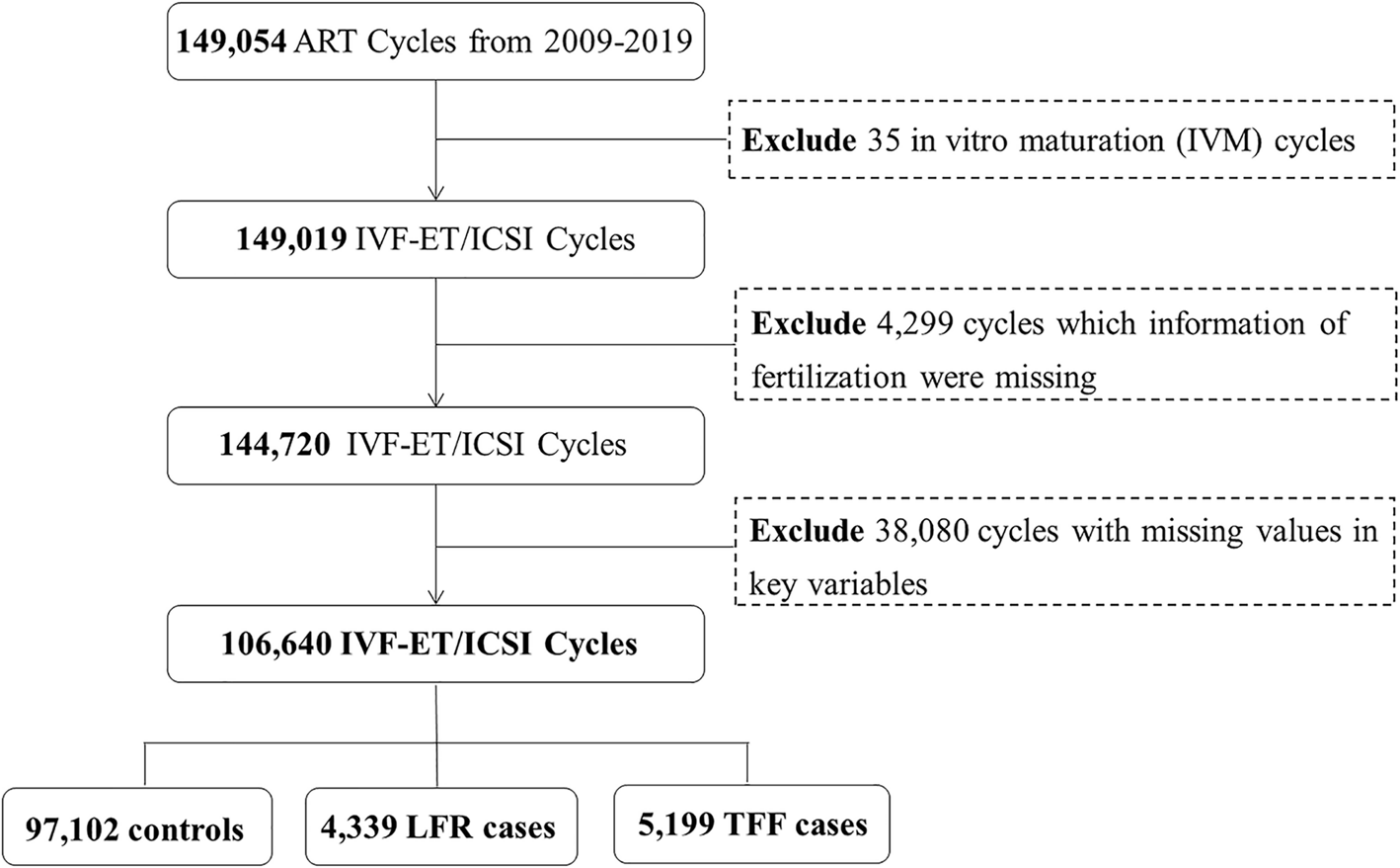
Flow chart of the cycle selection in this study. LFR: Low fertilization rate; TFF: Total fertilization failure

The retrospective data for this study were collected from medical records for IVF/ICSI cycles from 2009 to 2019 at the Center for Reproductive Medicine, Peking University Third Hospital, one of China's largest reproductive health centers. Detailed information on the database is described in our previous publication [17]. In total, 149,054 fresh embryo transfer cycles were initially included in this study. The following cycles were excluded: 35 in vitro maturation cycles, 4,299 cycles lacking information on fertilization outcomes, and 38,080 cycles with missing values for the critical variables. Therefore, 106,640 IVF/ICSI cycles were included in the subsequent analysis (Fig. 1).

This study was approved by the Ethics Committee of Peking University Third Hospital (No. IRB00006761-M2020004). Informed consent was waived because this was a data analysis with no personally identifiable information.

### Preprocessing of data

#### Data collection

Based on prior evidence from our previous analysis [21], we initially included 24 variables in this study. The variables were classified into three categories: 1) 13 female-related variables, including age, body mass index (BMI, kg/m^2^), parity, gravidity, genetic disorders, abnormal gestation history, infertility type, ART failure history, diminished ovarian reservation (DOR), and infertility factors, such as ovulatory disorders, uterine disorders, fallopian tube disorders, hyperprolactinemia, and endometriosis. 2) 5 male-related variables, including age, BMI, and sperm quality [normal, oligoasthenozoospermia (OAZ), severe OAZ, and azoospermia], which was determined by the WHO laboratory manual for the examination and processing of human semen, 5th ed. [22]. The sperm preparation and measurement methods during the duration of the study were consistent. 3) 6 variables related to ART treatment, including the controlled ovarian hyperstimulation (COH) protocol, number of oocytes retrieved (< 5, 5–20, > 20), antral follicle count (AFC), and insemination method (IVF or ICSI). Before constructing the model, the predictors were transformed into categorical variables. Detailed information on those variables is shown in Supplemental Table 1.

#### Variable definitions

The outcomes were defined according to the Chinese Society of Reproductive Medicine (CSRM) consensus on crucial indicators for quality control in ART clinical operation [23]. The fertilization rate (FR) of IVF was calculated as (the number of oocytes with two pronuclei/the number of all collected oocytes)*100. The FR of ICSI was calculated as (the number of oocytes with two pronuclei/the number of all collected oocytes in the MII period)*100. TFF was defined as a cycle resulting in no fertilized oocytes. An LFR was defined as a cycle with an FR < 25% [24, 25].

### Bayesian network model

The database was randomly divided into two datasets. Seventy-five percent of the cycles were randomly selected as a training dataset and used to construct a BN model, while remaining 25% were used as an external test dataset.

#### Bayesian network definition

BNs are classification algorithms based on machine learning that can be used to conduct causal reasoning and risk prediction analysis. A BN can offer several advantages over conventional regression-based methods. BNs are graphic representations that include DAGs and conditional probability tables (CPTs). A DAG includes nodes representing the variables in the network and directed edges (depicted as arrows between nodes) representing associations between the variables. The absence of an arrow between a pair of nodes implies independence between the variables. CPTs describe the direction of influence among variables and the degree of influence [26].

#### Variable selection

Predictors were selected based on prior knowledge provided by previous studies and the advice of clinical experts [17]. Before performing BN analysis, chi-square analysis was performed to estimate the potential association between each predictor and fertilization disorders, which could reduce the total number of variables to avoid overfitting the model to the data. As a result, 21 variables showed significantly different distributions between the control/LFR/TFF groups and were thus included in the subsequent BN analysis (Supplemental Table 2).

#### Structure learning

**Fig. 2:**
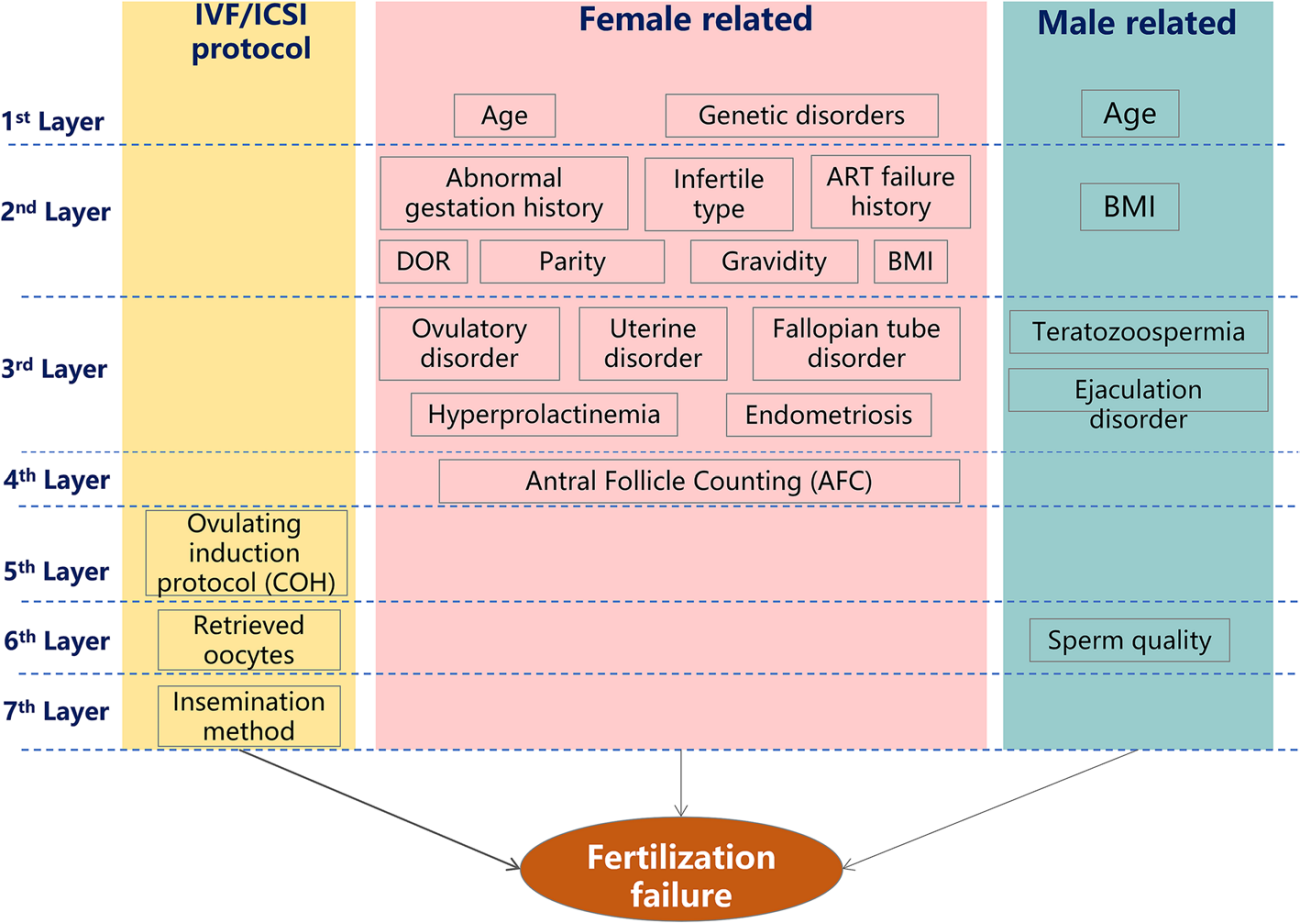
Theoretical model diagram of the Bayesian network model. Before the BN structure was established, a theoretical model diagram was developed according to prior knowledge from previous literature and clinical practice on the association between the selected variables. The variables were divided into seven hierarchical layers and were categorized into three subgroups

A theoretical model diagram of the BN model was developed according to prior knowledge from previous literature and clinical practice on the association between the selected variables experts [21]. As shown in Fig. 2, the variables were divided into seven hierarchical layers. We also provided a blacklist, which refers to a list of arcs in the blacklist that are never included in the network (e.g., the genetics factor cannot influence the female age. Therefore, the arc from “genetic factor” to “female age” is included the blacklist and is never included in the network during machine learning). The variables in the latter layer cannot influence the variables in the former layers, which means that the arcs cannot point from the latter layers to the former layers. In addition, female-related variables are considered not to be adjusted with male-related variables. We performed structure learning based on the Hill Climber Bayes Net method, which can add, delete, and reverse edges (arrows) as it searches through the feature space and terminates when an optimal model structure is achieved [27]. First, we included all 21 variables in BN structure learning model. Then, we excluded the variables that were not in the DAG or those that pointed to fertilization failure and relearned the DAG structure. Tenfold cross-validation was performed, and each model's log-likelihood posterior classification error was calculated to evaluate the two BN structures. A model with a lower log-likelihood posterior classification error is considered better.

### Performance of the model

The remaining 25% of the cycles were used to evaluate the external validity of the models. The BN model was assessed by its accuracy, receiver operating characteristic (ROC) curve, and corresponding area under curve (AUC). Accuracy was defined as the sum of true positive and true negative instances divided by the total number of instances. The ROC curve shows the model sensitivity and (1-specificity), while the AUC value refers to the ability of the risk prediction model to classify research objects correctly. The cutoff point of the ROC curve was also calculated to obtain the sensitivity and specificity of the risk prediction model. Moreover, a decision tree model analysis was performed to examine the robustness of the BN model.

All statistical tests were two-sided, and a *P value* < 0.05 was considered statistically significant. The data analysis in this study was performed using R (version 4.1.0). The development and validation of the prediction models were implemented by using a series of R packages, including “BNlearn”, “MASS”, “caret”, “ggplot2”, and "pROC", and other R Core Teams.

## Results

### Characteristics of cycles

Between 2009 and 2019, a total of 149,054 IVF/ICSI cycles were included. After excluding 35 in vitro maturation cycles, 4,299 cycles without fertilization information, and 38,080 cycles with missing values in key predictor variables, 106,640 cycles were included in the final analysis, including 97,102 controls, 4,339 LFR cases, and 5,199 TFF cases (Fig. 1). The basic characteristics of the cycles are shown in Table 1. A total of 34.1% of the cycles were older than 35 years old. A total of 59.0% of the cycles did not have a history of pregnancy, while 93.1% had no parity. The frequencies of women with a BMI greater than or equal to 25, fallopian tube disorders, uterine disorders, hyperprolactinemia, ovulatory disorders, ovarian surgery, diminished ovarian function, endometriosis, genetic disorders, and and abnormal gestational history were 29.8%, 21.4%, 7.5%, 13.1%, 0.3%, 9.7%, 5.5%, 0.9%, and 0.9%, respectively. For 4.3% of the cycles, the male partners were older than 45 years old. A total of 39.5% of the cycles had OAZ or severe OAZ, while 7.3% had azoospermia. The frequency of primary infertility was 54.9%, and the ART failure rate was 34.3%. In 12.6% of the cycles, less than five oocytes were retrieved.

**Table1:** Baseline characteristics of the study population

	**Characteristics**	**Levels**	**Overall (*N* = 106,640)**	
			**N**	%
**Female**	**Age (y)**	≤ 29	28,459	26.7
		30–34	41,852	39.2
		35–37	18,065	16.9
		38–40	10,577	9.9
		41–42	4118	3.9
		≥ 43	3569	3.5
	**BMI (kg/m**^**2**^**)**	18.5–24.0	66,463	62.3
		< 18.5	8423	7.9
		24.0–28	23,480	22.0
		≥ 28	8274	7.8
	**Gravidity**	0	62,692	59.0
		1	23,579	22.1
		≥ 2	20,099	18.8
	**Parity**	0	99,279	93.1
		≥ 1	7361	6.9
	**Fallopian tube disorders**		22,874	21.4
	**Uterine disorders**		8041	7.5
	**Hyperprolactinemia**		424	0.4
	**Ovulatory disorders**		13,917	13.1
	**Ovarian cyst surgery**		305	0.3
	**Diminished ovarian function**		10,312	9.7
	**Endometriosis**		5817	5.5
	**Genetic disorders**		977	0.9
	**Abnormal gestational history**		948	0.9
**Male**	**Age (y)**	≤ 45	102,106	95.7
		> 45	4534	4.3
	**BMI (kg/m**^**2**^**)**	< 18.5	1513	1.4
		18.5–24.0	37,240	34.9
		24.0–28.0	45,187	42.4
		≥ 28.0	22,700	21.3
	**Ejaculatory disorders**		149	0.1
	**Teratozoospermia**		4680	4.4
	**Sperm quality**	Normal	56,788	53.2
		OAZ	38,675	36.3
		Severe OAZ	3398	3.2
		Azoospermia	7779	7.3
**ART**	**Infertility type**	Primary	58,545	54.9
		Secondary	48,095	45.1
	**ART failure history**	No	70,040	65.7
		Yes	36,600	34.3
	**Ovulation induction protocol**	Stimulation cycle	101,349	95.0
		Minimal-stimulation cycle	4639	4.4
		Natural cycle	652	0.6
	**Antral follicle count**	> 12	36,479	34.2
		5–12	61,786	57.9
		< 5	8337	7.9
	**Number of oocytes retrieved**	≥ 20	15,770	14.8
		5–20	77,398	72.6
		< 5	13,472	12.6
	**Insemination method**	ICSI	47,137	44.2
		IVF	59,503	55.8

### Predictor selection

The associations between each predictive variable and fertilization disorders were initially explored by the chi-square test. As Supplemental Table 2 shows, female age significantly differed among the groups, and the frequency of an age ≥ 43 was significantly higher in the TFF group than in the control group. The frequencies of a history of uterine disorders, diminished ovarian function and endometriosis, natural cycles, an AFC < 5, primary infertility, and a male partner age ≥ 45 years were significantly higher in the TFF group than in the control group (all *Ps* < 0.05), implying that these variables may be more predictive than other variables. In multiple comparisons, the frequencies of an LFR did not show a significant difference between most of these characteristics. In addition, the frequencies of hyperprolactinemia, ovarian surgery, and ejaculation disorders were not significantly different among the three groups, so these three predictors were excluded from the subsequent BN analysis.

### Bayesian network model

Figure 2 shows the theoretical model diagram of the BN model, which was determined by prior knowledge [17]. The predictors were divided into three categories: female-related, male-related, and treatment-related variables. The variables were organized into seven hierarchical layers. Theoretically, the former layer could not be causally influenced by the latter layer.

**Fig. 3:**
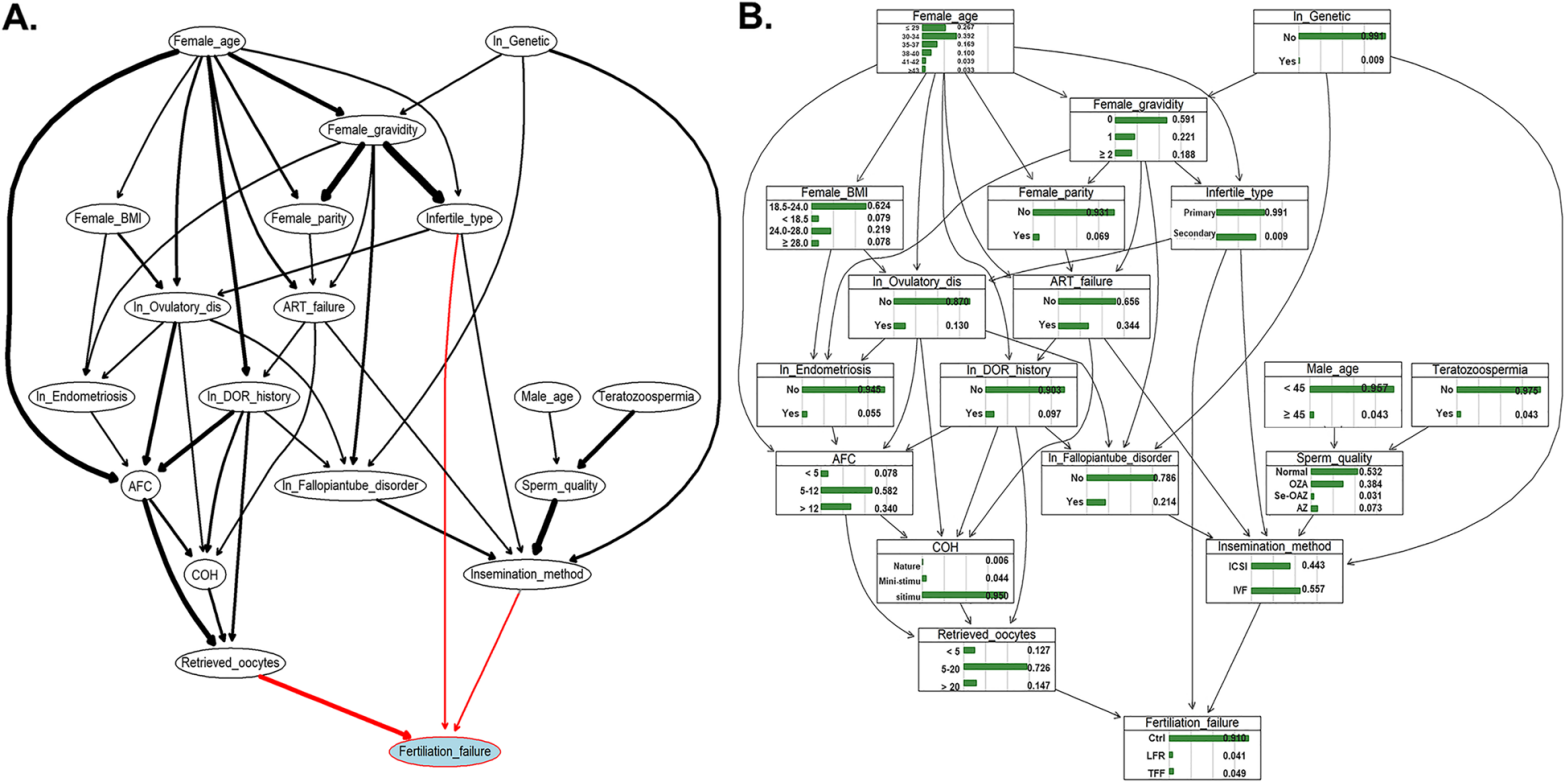
Directed acyclic graphs of the Bayesian network model. *Abbreviations:* “In_” refers to “Infertility factor_”; In_Ovulatory_dis: Infertility factor_ovulatory disorder; ART_failure: ART failure history; AFC: Antral Follicle Counting; COH: Ovulating induction protocol

The BN model analysis based on a theoretical diagram graph was subsequently performed. Initially, all 21 variables were included. As Supplemental Figure 1 shows, male BMI, a history of abnormal gestations, and uterine disorders were not associated with fertilization failure. Therefore, we excluded these three variables and reconstructed the BN model using the remaining 18 variables. Tenfold cross-validations showed that the BN model including the remaining 18 variables had less posterior classification error than the BN model, including all predictors (Supplemental Figure 2). The DAG of the networks, including only 18 variables, is shown in Fig. 3. The directions of arcs refer to the causal relationships, while the thicknesses represent the strength of the association between variables. As Fig. 3A depicts, female age and genetic disorders influenced several “mediating” variables, thus influencing the fertilization rate. Male-related variables mainly influenced fertilization disorders by mediating sperm quality. The number of retrieved oocytes, insemination methods, and infertility type could directly influence the probability of LFR/TFF. The number of retrieved oocytes had a stronger association than the other two predictors, implying that this is the most predictive variables of TFF. The DAG with proportions of each category of variables is shown in Fig. 3B.

**Fig. 4:**
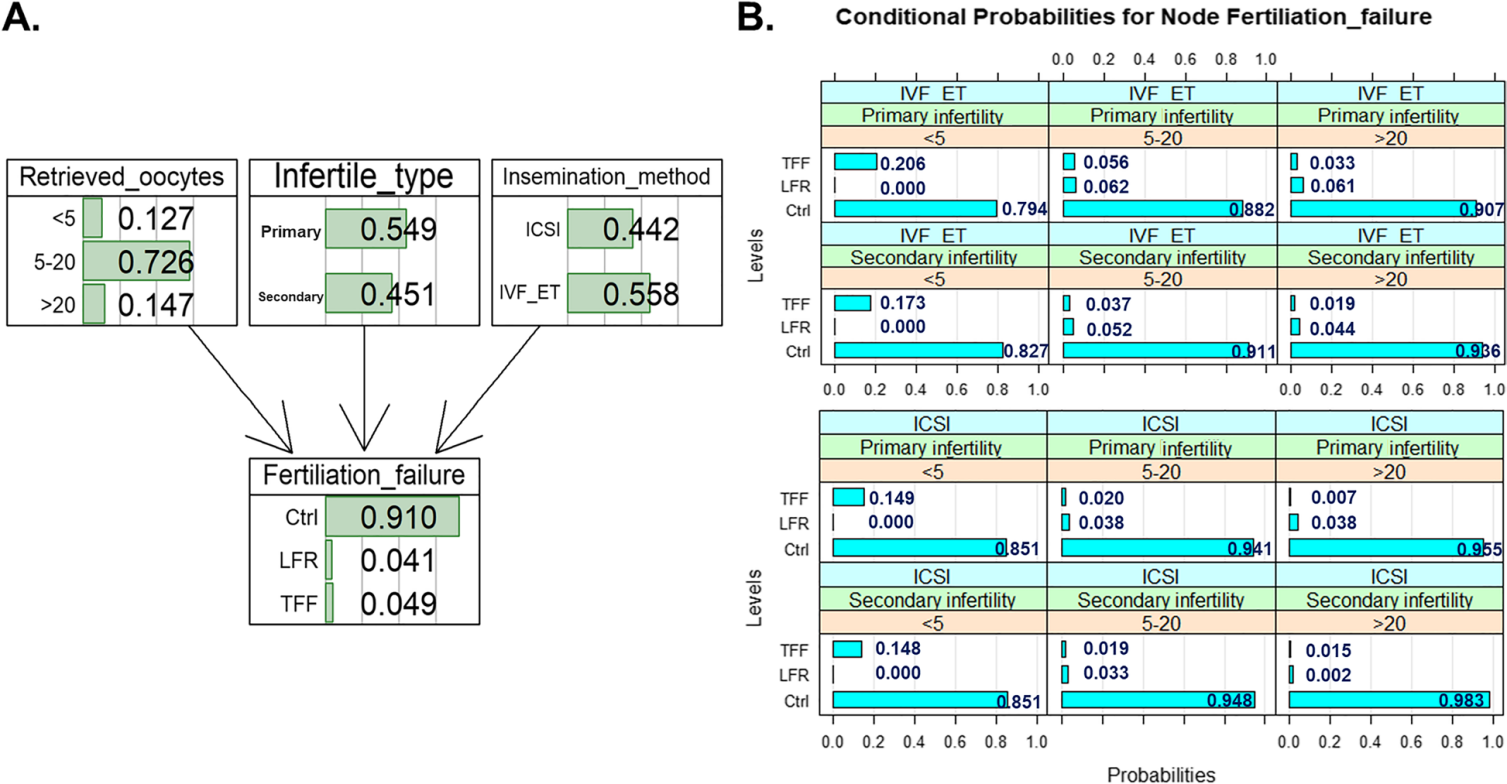
Conditional probabilities for node fertilization failure

The CPT conditional probability table directly pointing to fertilization failure was further provided by the BN model (Fig. 4A). As Fig. 4B shows, compared with a number of retrieved oocytes equal to five or more, the probability of TFF was higher when the number of retrieved oocytes was < 5. Primary infertility showed a higher probability of TFF/LFR than secondary infertility. The IVF-ET insemination method had a higher probability of TFF/LFR than the ICSI insemination method. The probability of TFF reached its highest value of 20.6% when a patient had primary infertility, the number of retrieved oocytes was less than five and when a patient had undergone an IVF cycle.

### Validation of the prediction model

**Fig. 5:**
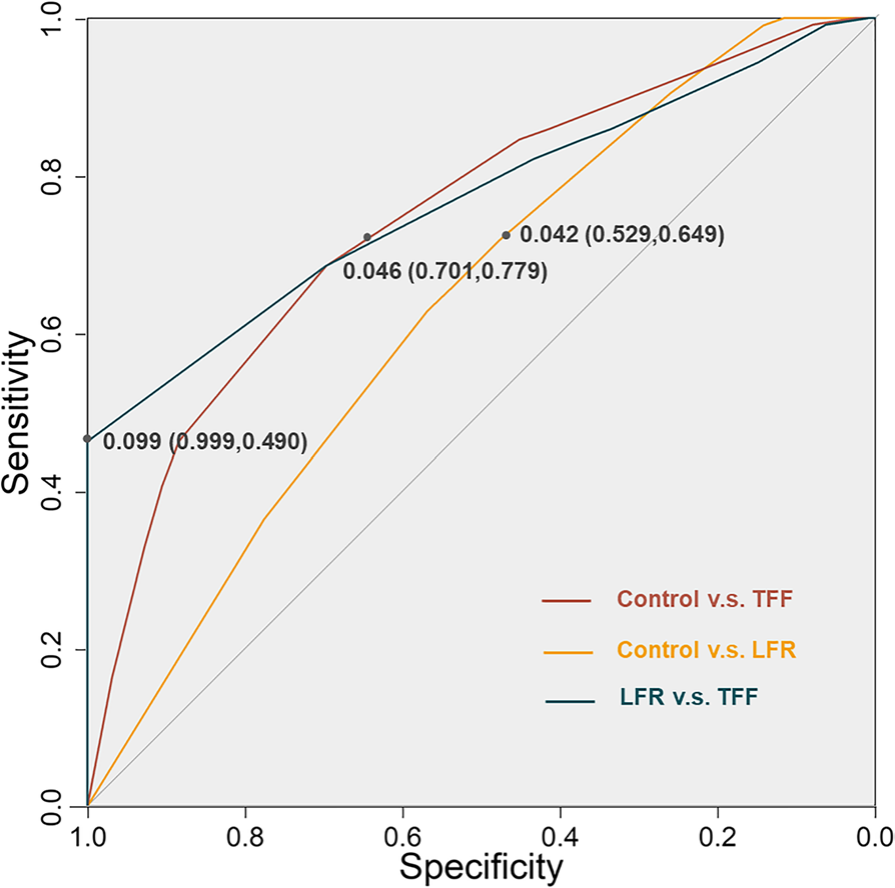
Receiver operating characteristic curve of the Bayesian network model. The best threshold points and values were shown in each curve. The specificities and sensitivities at the threshold point were shown in the brackets. LFR: low fertilization rate; TFF: total fertilization failure

The model was validated in the remaining 25% of the dataset. As a result, the prediction accuracy of the model was 91.3%. The ROC curves and AUCs are depicted in Fig. 5. The AUC of the control versus TFF groups was 0.779 (95% CI: 0.766–0.791), with a sensitivity of 71.2% and specificity of 70.1%. The AUC of the control versus LFR groups was 0.619 (95% CI: 0.605–0.634), with a sensitivity of 64.9% and specificity of 52.6%. Moreover, the AUC of the TFF versus LFR groups was 0.807 (95% CI: 0.790–0.824), with a sensitivity of 49.0% and specificity of 99.0%.

## Discussion

Fertilization is the most vital step during ART treatment, while failure or low fertilization could directly lead to unsuccessful outcomes, even ART treatment failure. In this study, based on real-world data that includes 106,640 IVF/ICSI cycles, we, for the first time, report a predictive model for IVF-related outcomes (fertilization disorders) derived from BN analysis, which is more accurate and flexible for dynamic real-world data than traditional regression models. The final BN model included nonmodifiable/historical variables (such as female age, gravity, parity, and a history of reproductive disease) and modifiable variables (such as female BMI, COH methods, and insemination methods). The former nonmodifiable variables help extrapolate future predictions based on the current trajectory, while the latter could help to provide clues for decision-making and modifying the protocols during ART medical treatment.

Currently, the fields of clinical medicine are undergoing a data revolution. Large volumes of medical records are being converted to electronic formats, which leads to remarkable growth in the data collected by health registries and during clinical studies, thus providing opportunities to make risk prediction and intervention selection more precise based on “big real-world data”. Bayesian statistics have been rapidly developed to solve real-world data problems and have now permeated all the major areas of medical statistics, including clinical trials, epidemiology, predictive modeling, and decision-making [28]. BN analysis could provide a natural way to represent the uncertainties involved in medicine when dealing with diagnosis, treatment selection, planning, and prognosis prediction [19]. Recently, various studies have reported BN-based predictive models and decision support systems in the medical field. One study derived a new Bayesian network-based risk stratification model for the prediction of short-term and long-term mortality in patients with left ventricular assist devices, with accuracies of 95%, 90%, 90%, 83%, and 78%, and ROCs of 91%, 82%, 82%, 80% and 81% respectively at each endpoints postimplantation (30 day, 90 day, 6 months, 1 year, and 2 years), which were higher than those of traditional conventional predictive models [27]. A study performed a BN analysis of the probabilistic relationships between various obesity phenotypes and cardiovascular disease risk based on 6276 individuals within the Chinese population and showed that the probability of cardiovascular disease risk was influenced by age and sex [17]. Bayesian network structures have also been developed and validated to predict tumor risk, recurrence, and survival [29]. These previous studies indicate that Bayesian models can reliably represent the complex causal relationships of multiple variables with clinical outcomes. However, the BN model has not yet been used in the field of reproductive health. Moreover, although some studies have reported predictive models for fertilization disorders based on traditional statistical analysis, no model has been widely used in clinical practice [16]. One European study reported that the number of retrieved oocytes, female smoking, and nontubal factor infertility were predictors of TFF [14]. Another study reported that male age, the number of IVF cycles, the indication for IVF, and the prewash total motile sperm count were predictors for TFF with an AUC of 0.75 [15]. Our previous study developed a traditional logistic analysis-based predictive model for TFF/LFR based on clinical variables. The AUC for the TFF group was 0.743 (95% CI: 0.729–0.757), with a sensitivity of 66.1% and specificity of 70.3% [21].

We reported a BN predictive model for fertilization failure in the present study. The AUC for the TFF group using the BN model was 0.779 (95% CI: 0.766–0.791), which was higher than that in previous studies. The sensitivity and specificity of the BN model for the TFF group were 71.2% and 70.1%, respectively. The sensitivity was higher than that of the traditional model, implying that the model could distinguish TFF cases from controls more easily and sensitively. Notably, the AUC of the TFF versus LFR groups in this study reached 0.807 (95% CI: 0.790–0.824), indicating that this BN model had good performance in distinguishing TFF from LFR, which could help clinicians identify patients who may have TFF from those with a LFR. The BN model identified three key variables, including infertility type, insemination method, and the number of oocytes retrieved, that were the most crucial predictors for TFF/LRF and directly impacted the probability of fertilization disorders. For a couple diagnosed with primary infertility, and less than 5 retrieved oocytes (nonmodified variables), the probability for TFF was 20.6% when they underwent IVF-ET insemination. Nevertheless, the probability could decrease to 14.9% when they undergo ICSI. This model is meaningful in assisting clinicians in a “step-by-step” manner to modify their detailed protocols to decrease the probability of TFF.

Most traditional regression-based models can only show how each variable relates to the outcomes. Nevertheless, these models do not perform well in determining the predictors' interactions. In contrast to traditional statistical methods, which comprise weighted combinations of independent variables, BNs provide the advantages of a rigorous probabilistic framework to perform inference for multiple variables and an intuitive representation that clinicians can easily interpret. In this study, we found that although several variables did not show direct associations with fertilization failure, they could influence other vital variables and form complex networks, thus affecting fertilization failure. For example, in the current BN model, female age could influence factors such as the AFC, BMI, parity, gravidity, and female disease history. These variables further influenced the key variables, such as the number of retrieved oocytes and insemination methods during treatment, thus contributing to the probability of fertilization failures. The “step-by-step” network provides a more accurate and detailed description and improves the performance of the patient decision-making process compared to the “black-box” risk scores, which can only take limited numbers of variables into account and have difficulty representing the complexity of the occurrence of LFR/TFF.

Missing information for several variables frequently occurs among patient records, and traditional regression-based models do not solve this problem. BN models can intuitively present relationships between predictors, evaluate potential possibilities for outcomes layer by layer based on some given predictors, fill missing values and solve “uncertainties” by calculating conditional possibilities based on a parent node. Therefore, BN models have already been translated into clinical practice [30, 31]. For example, the Pittsburgh Cervical Cancer Screening Model, a dynamic Bayesian network assessing the risk of cervical precancer and invasive cancer, has been established. It was constructed based on expert knowledge and follow-up data collected over 13 years [32]. BN models also performed better in diagnosing dementia, AD, and mild cognitive impairment (MCI) compared to most other well-known classifiers/models [31]. Therefore, based on the BN model validated in this study, we plan to develop an online application available to clinicians and patients, that could easily interpret the predictors and key decision points of fertilization failure along the continuum of a patient’s clinical course. The application will be integrated with electronic health records to compute patients' probabilities of TFF/LFR based on the most current clinical data available. An additional feature will allow reproductive clinicians to customize the decision support tool according to each patient's unique conditions.

Some limitations need to be addressed before any potential extrapolation of the findings. First, our study was based on a single-center analysis. Although this center is one of the largest reproductive health centers in China, serving infertile couples from all 31 provinces in China, the BN model constructed by using the single-center clinical data in this study should be further verified by external data. Second, since our data were automatically extracted from the computer-based patient record system, the detailed IVF laboratory parameters of sperm and oocytes used for insemination were unavailable; thus, the impact of sperm and oocyte parameters on fertilization outcomes was not evaluated in this study. Third, based on the center’s data sharing & access regulations, any personally identifiable information (such as name and ID card) should be excluded from the dataset used for scientific research. So, we could only obtain data based on IVF cycles. The data used in this study were per cycle, not per couple or patient, so the BN model might not apply to patients with recurrent fertilization disorders. Further research is needed to explore such predictive factors or models for chronic fertilization disorders and consider the influence of repeated measurement and recurrent fertilization disorders. Fourth, since most of the predictors used in this study were known factors, we did not provide new predictors for TFF/LFR. However, the primary purpose of our study was to develop a predictive model with better performance and fit for real-world data based on known clinical factors rather than to capture new risk factors for TFF/LFR. Fifth, rather than giving a specific cutoff value as in traditional regression models, in the BN model, the decision-making meant that we could compare possibilities of TFF/LFR in different protocols and then choose the optimal protocol with the lowest predicted probability of TFF/LFR. Finally, the BN model performed well in distingguishing TFF patients from LFR patients and controls. Identifying TFF patients is of great concern for clinicians and embryologists. However, the model did not perform as well in distinguishing LFR from controls. Therefore, the BN model should be further optimized to better distinguish LFR group from controls by using more sensitive predictors in further research. In addition, we deleted cycles with missing values rather than filling the missing values by using some statistical approaches. However, we compared distributions of the critical variables between excluded (incomplete data) and included (complete data) data. The frequencies of key variables such as the age of the partners, number of retrieved oocytes, insemination method, and infertility type, which directly influenced fertilization in the BN model, were comparable between the included and excluded cycles. This implies that the missing data were likely to be missing at random, and deleting these missing values may not have introduced much bias (Supplementary Table 3).

## Conclusion

In conclusion, in this study, we developed a predictive model based on the BN learning method and revealed that several clinical factors could form a network, thus indirectly or directly influencing the occurrence of LFR/TFF. The insemination method, infertility type, and number of retrieved oocytes were direct predictors of TFF/LFR. The BN model provided relatively good performance in distinguishing the TFF group from controls and LFR groups, but the performance in distinguishing the LFR group from controls needs improvement. The model could be used to build clinical decision support systems to predict fertilization disorders and further provide new evidence for the power of the BN model and its wider use in modeling and the analysis of medical data, especially when the analysis concerns areas of diagnostic or prognostic uncertainty and a sizeable number of variables are involved in the process.

## Supplementary Information

**Additional file 1:** **Table S1.** The information of involved variables. **Table S2.** Predictor selection. **Table S3.** Comparison between included and excluded data. **Figure S1.** Bayesian Network model based on all predictors. **Figure S2.** Tenfold ten-cross validation.

## Data Availability

The data underlying this article will be shared upon reasonable request to the corresponding author.
