# Supplementary material for: A Bayesian network model for prediction of low or failed fertilization in assisted reproductive technology based on a large clinical real-world data
Source: Reprod Biol Endocrinol. 2023 Jan 26;21:8. doi: 10.1186/s12958-023-01065-x (PMC9878771; doi:10.1186/s12958-023-01065-x)

**A Bayesian Network Model for prediction of low or failed fertilization in assisted reproductive technology based on a large clinical real-world data**

**Running title:** Bayesian network model for fertilization failure in IVF

Tian Tian ^1,2,3,4^, Fei Kong ^1,2,3,4^, Rui Yang ^1,2,3,4^, Xiaoyu Long ^1,2,3,4^, Lixue Chen ^1,2,3,4^, Ming Li ^1,2,3,4^, Qin Li ^1,2,3,4^, Yongxiu Hao ^1,2,3,4^, Yangbo He ^5^, Yunjun Zhang ^6^, Rong Li ^1,2,3,4^, Yuanyuan Wang ^1,2,3,4^ *, Jie Qiao ^1,2,3,4,7,8^*

1. Center for Reproductive Medicine, Department of Obstetrics and Gynecology, Peking University Third Hospital
2. National Clinical Research Center for Obstetrics and Gynecology (Peking University Third Hospital)
3. Key Laboratory of Assisted Reproduction (Peking University), Ministry of Education
4. Beijing Key Laboratory of Reproductive Endocrinology and Assisted Reproductive Technology (Peking University Third Hospital)
5. School of Mathematical Sciences, LMAM, LMEQF, and Center of Statistical Science, Peking University, Beijing, China
6. School of public health, Peking University, Beijing, China
7. Beijing Advanced Innovation Center for Genomics, Beijing, China
8. Peking-Tsinghua Center for Life Sciences, Peking University, Beijing, China

**Correspondence:**

Jie Qiao, Ph.D., M.D., Department of Obstetrics and Gynecology, Peking University Third Hospital, China (e-mail: jie.qiao@263.net).

Yuanyuan Wang, Ph.D., Department of Obstetrics and Gynecology, Peking University Third Hospital, China (e-mail: yyuanwang@163.com).

**Table S1** The information of involved variables.

|  | **Predictors** | **Description** |
| --- | --- | --- |
| **Female** |  |  |
| **1** | Female age (years) | “≤29”=0; “30-34”=1; “35-37”=2; “38-40”=3; “41-42”=4; “≥43”=5 |
| **2** | Female BMI (kg/m^2^) | “18.5-24.0”= 0; “< 18.5”=1; “24.0-28.0”=2;  "≥ 28" = 3 |
| **3** | Gravidity | “0”=0; “1”=1; “≥2”=2 |
| **4** | Parity | “0”=0; “≥1”=1 |
| **5** | Fallopian tube disorders | "No" = 0; "Yes" = 1 |
| **6** | Uterine disorders | "No" = 0; "Yes" = 1 |
| **7** | Hyperprolactinemia | "No" = 0; "Yes" = 1 |
| **8** | Ovulatory disorders | "No" = 0; "Yes" = 1 |
| **9** | Ovarian cyst surgery | "No" = 0; "Yes" = 1 |
| **10** | DOR_history | "No" = 0; "Yes" = 1 |
| **11** | Endometriosis | "No" = 0; "Yes" = 1 |
| **12** | Genetic disorders | "No" = 0; "Yes" = 1 |
| **13** | Abnormal gestational history | "No" = 0; "Yes" = 1 |
| **Male** |  |  |
| **14** | Male age | "< 45" = 0；"≥ 45" = 1 |
| **15** | Male BMI (kg/m^2^) | "18.5-24.0" = 0; "< 18.5" = 1; "24.0-28.0" = 2; "≥ 28" = 3 |
| **16** | Ejaculatory dysfunction | "No" = 0; "Yes" = 1 |
| **17** | Teratozoospermia | "No" = 0; "Yes" = 1 |
| **18** | Sperm quality | "Normal" = 0; "Oligoaasthenozoospermia" = 1; "severe oligoaasthenozoospermia" = 2; "azoospermia" = 3 |
| **ART Treatment-related** | |  |
| **19** | Infertility type | "Secondary" = 0; "Primary" = 1 |
| **20** | ART failure history | "No" = 0; "Yes" = 1 |
| **21** | Ovulating induction protocol | "Stimulation cycle" = 0; "Minimal-stimulation cycle" = 1; "Natural cycle" = 2 |
| **22** | Antral follicle count | ">12" = 0; "5-12" = 1; "<5" = 2 |
| **23** | Number of oocytes retrieved | ">20" = 0; "5-20" = 1; "<5" = 2 |
| **24** | Insemination method | "ICSI" = 0; "IVF" =1 |

# Table S2 Predictor selection

|  |  | **Ctrl** | **LFR** | **TFF** | **χ^2^** | ***P*** |
| --- | --- | --- | --- | --- | --- | --- |
|  |  | **N = 97,102** | **N = 4,339** | **N = 5,199** |  |  |
| Female age | ≤29 | 26244 (27.0) | 1221 (28.1) | 994 (19.1) | 1706.4 | <0.001 |
|  | 30-34 | 38326 (39.5) | 1752 (40.4) | 1774 (34.1) |  |  |
|  | 35-37 | 16471 (17.0) | 732 (16.9) | 862 (16.6) |  |  |
|  | 38-40 | 9415 (9.7) | 402 (9.3) | 760 (14.6) |  |  |
|  | 41-42 | 3619 (3.7) | 126 (2.9) | 373 (7.2) |  |  |
|  | ≥43 | 3027 (3.1) | 106 (2.4) | 436 (8.4) |  |  |
| Female BMI (kg/m2) | 18.5-24.0 | 60632 (62.4) | 2680 (61.8) | 3151 (60.6) | 13.197 | 0.012 |
|  | < 18.5 | 7706 (7.9) | 325 (7.5) | 392 (7.5) |  |  |
|  | 24.0-28 | 21297 (21.9) | 968 (22.3) | 1215 (23.4) |  |  |
|  | ≥ 28 | 7467 (7.7) | 366 (8.4) | 1. (8.5) |  |  |
| Female gravidity | 0 | 57073 (58.8) | 2713 (62.5) | 3176 (61.1) | 13.197 | 0.001 |
|  | 1 | 21560 (22.2) | 916 (21.1) | 1103 (21.2) |  |  |
|  | 2 | 18469 (19.0) | 710 (16.4) | 920 (17.7) |  |  |
| Female parity | 0 | 90445 (93.1) | 4077 (94.0) | 4757 (91.5) | 78.907 | <0.001 |
|  | 1 | 6657 (6.9) | 262 (6.0) | 442 (8.5) |  |  |
| In_Fallopian tube disorders | No | 76240 (78.5) | 3411 (78.6) | 4140 (80.0) | 7.260 | 0.026 |
|  | Yes | 20862 (21.5) | 928 (21.4) | 1029 (20.0) |  |  |
| In_Uterine disorders | No | 89894 (92.6) | 3995 (92.1) | 4710 (90.6) | 60.664 | <0.001 |
|  | Yes | 7208 (7.4) | 344 (7.9) | 489 (9.4) |  |  |
| In_Hyperprolactinemia | No | 96716 (99.6) | 4323 (99.6) | 5177 (99.6) | 0.17 | 0.915 |
|  | Yes | 386 (0.4) | 16 (0.4) | 22 (0.4) |  |  |
| In_Ovulatory disorders | No | 84330 (86.8) | 3761 (86.7) | 4632 (89.1) | 43.537 | <0.001 |
|  | Yes | 12772 (13.2) | 578 (13.3) | 567 (10.9) |  |  |
| In_Ovarian cyst surgery | No | 96823 (99.7) | 4326 (99.7) | 5186 (99.7) | 1.237 | 0.874 |
|  | Yes | 279 (0.3) | 13 (0.3) | 13 (0.3) |  |  |
| In_DOR history | No | 88326 (91.0) | 4028 (92.8) | 3974 (76.4) | 2235.4 | <0.001 |
|  | Yes | 8776 (9.0) | 311 (7.2) | 1225 (23.6) |  |  |
| In_Endometriosis | No | 91915 (94.7) | 4079 (94.0) | 4829 (92.9) | 60.015 | <0.001 |
|  | Yes | 5187 (5.3) | 260 (6.0) | 370 (7.1) |  |  |
| In_Genetic disorders | No | 96153 (99.0) | 4323 (99.6) | 5187 (99.8) | 53.688 | <0.001 |
|  | Yes | 949 (1.0) | 16 (0.4) | 12 (0.2) |  |  |
| In_Abnormal_gestationhistory | No | 96212 (99.1) | 4308 (99.3) | 5172 (99.5) | 8.592 | 0.006 |
|  | Yes | 890 (0.9) | 31 (0.7) | 27 (0.5) |  |  |
| Male age | ≤ 45 | 93144 (95.9) | 4182 (96.4) | 4780 (91.9) | 399.72 | <0.001 |
|  | > 45 | 3958 (4.1) | 157 (3.6) | 419 (8.1) |  |  |
| Male BMI (kg/m2) | 18.5-24.0 | 33966 (35.0) | 1483 (34.2) | 1791 (34.4) | 13.898 | 0.041 |
|  | < 18.5 | 1392 (1.4) | 62 (1.4) | 59 (1.1) |  |  |
|  | 24-28 | 41045 (42.3) | 1849 (42.6) | 2293 (44.1) |  |  |
|  | ≥ 28.0 | 20699 (21.3) | 945 (21.8) | 1056 (20.3) |  |  |
| Ejaculation disorders | No | 96960 (99.9) | 4335 (99.9) | 5196 (99.9) | 3.63 | 0.163 |
|  | Yes | 142 (0.1) | 4 (0.1) | 3 (0.1) |  |  |
| Teratozoospermia | No | 92860 (95.7) | 4116 (94.9) | 4971 (95.6) | 5.869 | 0.053 |
|  | Yes | 4242 (4.3) | 223 (5.1) | 228 (4.4) |  |  |
| Sperm quality | Normal | 51479 (53.0) | 2320 (53.5) | 2989 (57.5) | 202.2 | <0.001 |
|  | OAZ | 35241 (36.3) | 1518 (35.0) | 1916 (36.9) |  |  |
|  | Severe OAZ | 3137 (3.2) | 162 (3.7) | 99 (1.9) |  |  |
|  | Azoospermia | 7245 (7.5) | 339 (7.8) | 195 (3.8) |  |  |
| Infertility type | Primary | 53055 (54.6) | 2550 (58.8) | 2940 (56.5) | 40.479 | <0.001 |
|  | Secondary | 44047 (45.4) | 1789 (41.2) | 2259 (43.5) |  |  |
| ART failure history | No | 64088 (66.0) | 2821 (65.0) | 3131 (60.2) |  | <0.001 |
|  | Yes | 33014 (34.0) | 1518 (35.0) | 2068 (39.8) |  |  |
| Ovulation induction protocol | Natural cycle | 547 (0.6) | 16 (0.4) | 89 (1.7) | 2741.800 | <0.001 |
|  | Minimal-stimulation | 3860 (4.0) | 111 (2.6) | 668 (12.8) |  |  |
|  | Stimulation cycle | 92695 (95.5) | 4212 (97.1) | 4442 (85.4) |  |  |
| Antral follicle count | <5 | 7051 (7.3) | 226 (5.2) | 1098 (21.1) |  |  |
|  | 5-12 | 56223 (57.9) | 2589 (59.7) | 2974 (57.2) |  |  |
|  | >12 | 33828 (34.8) | 1524 (35.1) | 1127 (21.7) |  |  |
| Number of oocytes retrieved | <5 | 11146 (11.5) | 1 (0.0) | 2325 (44.7) |  | <0.001 |
|  | 5-20 | 71081 (73.2) | 3688 (85.0) | 2629 (50.6) |  |  |
|  | >20 | 14875 (15.3) | 650 (15.0) | 245 (4.7) |  |  |
| Insemination method | ICSI | 44215 (45.5) | 1502 (34.6) | 1420 (27.3) | 997.28 | <0.001 |
|  | IVF | 52887 (54.5) | 2837 (65.4) | 3779 (72.7) |  |  |

Note: In_ refers to “Infertility factor_”

# Table S3. Comparison between included and excluded data

| **Characteristics** | **Levels** | **Complete data (N=106,640)** | | **Incomplete data (n= 38080)** | | **χ^2^** | ***P*** |
| --- | --- | --- | --- | --- | --- | --- | --- |
|  |  | **N** | **%** | **N** | **%** |  |  |
|  |  |  |  |  |  |  |  |
| **Age (y)** | ≤29 | 28459 | 26.7 | 10030 | 26.4 | 11.50 | 0.070 |
|  | 30-34 | 41852 | 39.2 | 14912 | 39.2 |  |  |
|  | 35-37 | 18065 | 16.9 | 6420 | 16.8 |  |  |
|  | 38-40 | 10577 | 9.9 | 3790 | 10 |  |  |
|  | 41-42 | 4118 | 3.9 | 1490 | 3.9 |  |  |
|  | ≥43 | 3569 | 3.5 | 1407 | 3.7 |  |  |
| **BMI (kg/m^2^)** | 18.5-24.0 | 66463 | 62.3 | 21269 | 63.1 | 11.11 | 0.012 |
|  | < 18.5 | 8423 | 7.9 | 2664 | 7.9 |  |  |
|  | 24.0-28 | 23480 | 22 | 7256 | 21.5 |  |  |
|  | ≥ 28 | 8274 | 7.8 | 2509 | 7.4 |  |  |
| **Gravidity** | 0 | 62692 | 59 | 22500 | 60 | 13.87 | 0.001 |
|  | 1 | 23579 | 22.1 | 8090 | 21.6 |  |  |
|  | ≥2 | 20099 | 18.8 | 6887 | 18.4 |  |  |
|  |  |  |  |  |  |  |  |
| **Fallopian tube disorders** | | 22874 | 21.4 | 6683 | 17.6 | 262.6 | < 0.001 |
| **Uterine disorders** | | 8041 | 7.5 | 3415 | 9 | 78.46 | < 0.001 |
| **Hyperprolactinemia** | | 424 | 0.4 | 115 | 0.3 | 5.728 | 0.017 |
| **Ovulatory disorders** | | 13917 | 13.1 | 3919 | 10.3 | 197.67 | < 0.001 |
| **Ovarian cyst surgery** | | 305 | 0.3 | 207 | 0.5 | 52.811 | < 0.001 |
| **Diminished ovarian function** | | 10312 | 9.7 | 3769 | 9.9 | 1.656 | 0.198 |
| **Endometriosis** | | 5817 | 5.5 | 3190 | 8.4 | 405.99 | < 0.001 |
| **Genetic disorders** | | 977 | 0.9 | 284 | 0.7 | 8.564 | 0.003 |
| **Abnormal gestational history** | | 948 | 0.9 | 305 |  | 2.533 | 0.111 |
|  |  |  |  |  |  |  |  |
| **Age (y)** | ≤45 | 102106 | 95.7 | 35979 | 95.6 | 0.789 | 0.374 |
|  | > 45 | 4534 | 4.3 | 1640 | 4.4 |  |  |
| **BMI (kg/m^2^)** | < 18.5 | 1513 | 1.4 | 382 | 1.4 | 13.7 | 0.003 |
|  | 18.5-24.0 | 37240 | 34.9 | 9137 | 34.5 |  |  |
|  | 24.0-28.0 | 45187 | 42.4 | 11519 | 43.5 |  |  |
|  | ≥ 28.0 | 22700 | 21.3 | 5428 | 20.5 |  |  |
| **Ejaculatory disorders** | | 149 | 0.1 | 46 | 0.1 | 0.747 | 0.387 |
| **Teratozoospermia** | | 4680 | 4.4 | 912 | 2.4 | 300.23 | < 0.001 |
| **Sperm quality** | Normal | 56788 | 53.2 | 20290 | 53.4 | 2.184 | 0.535 |
|  | OAZ | 38675 | 36.3 | 13800 | 26.3 |  |  |
|  | Severe OAZ | 3398 | 3.2 | 1218 | 3.2 |  |  |
|  | Azoospermia | 7779 | 7.3 | 2682 | 7.1 |  |  |
|  |  |  |  |  |  |  |  |
| **Infertility type** | Primary | 58545 | 54.9 | 20741 | 54.6 | 1.25 | 0.263 |
|  | Secondary | 48095 | 45.1 | 17269 | 45.4 |  |  |
| **ART failure history** | No | 70040 | 65.7 | 25096 | 66 | 1.126 | 0.289 |
|  | Yes | 36600 | 34.3 | 12940 | 34 |  |  |
| **Ovulation induction protocols** | Stimulation cycle | 101349 | 95 | 36053 | 94.9 | 1.09 | 0.580 |
|  | Minimal-stimulation cycle | 4639 | 4.4 | 1672 | 4.4 |  |  |
|  | Natural cycle | 652 | 0.6 | 249 | 0.7 |  |  |
|  |  |  |  |  |  |  |  |
| **Antral follicle count** | >12 | 36479 | 34.2 | 3306 | 34.3 | 0.161 | 0.923 |
|  | 5-12 | 61786 | 57.9 | 5580 | 57.8 |  |  |
|  | < 5 | 8337 | 7.9 | 765 | 7.9 |  |  |
|  |  |  |  |  |  |  |  |
| **Number of oocytes retrieved** | ≥ 20 | 15770 | 14.8 | 5668 | 14.9 | 0.211 | 0.900 |
|  | 5-20 | 77398 | 72.6 | 27661 | 72.5 |  |  |
|  | < 5 | 13472 | 12.6 | 4792 | 12.7 |  |  |
|  |  |  |  |  |  |  |  |
| **Insemination method** | ICSI | 47137 | 44.2 | 16697 | 43.9 | 1.044 | 0.307 |
|  | IVF | 59503 | 55.8 | 21338 | 57.1 |  |  |

# Figure S1 Bayesian Network model based on all predictors


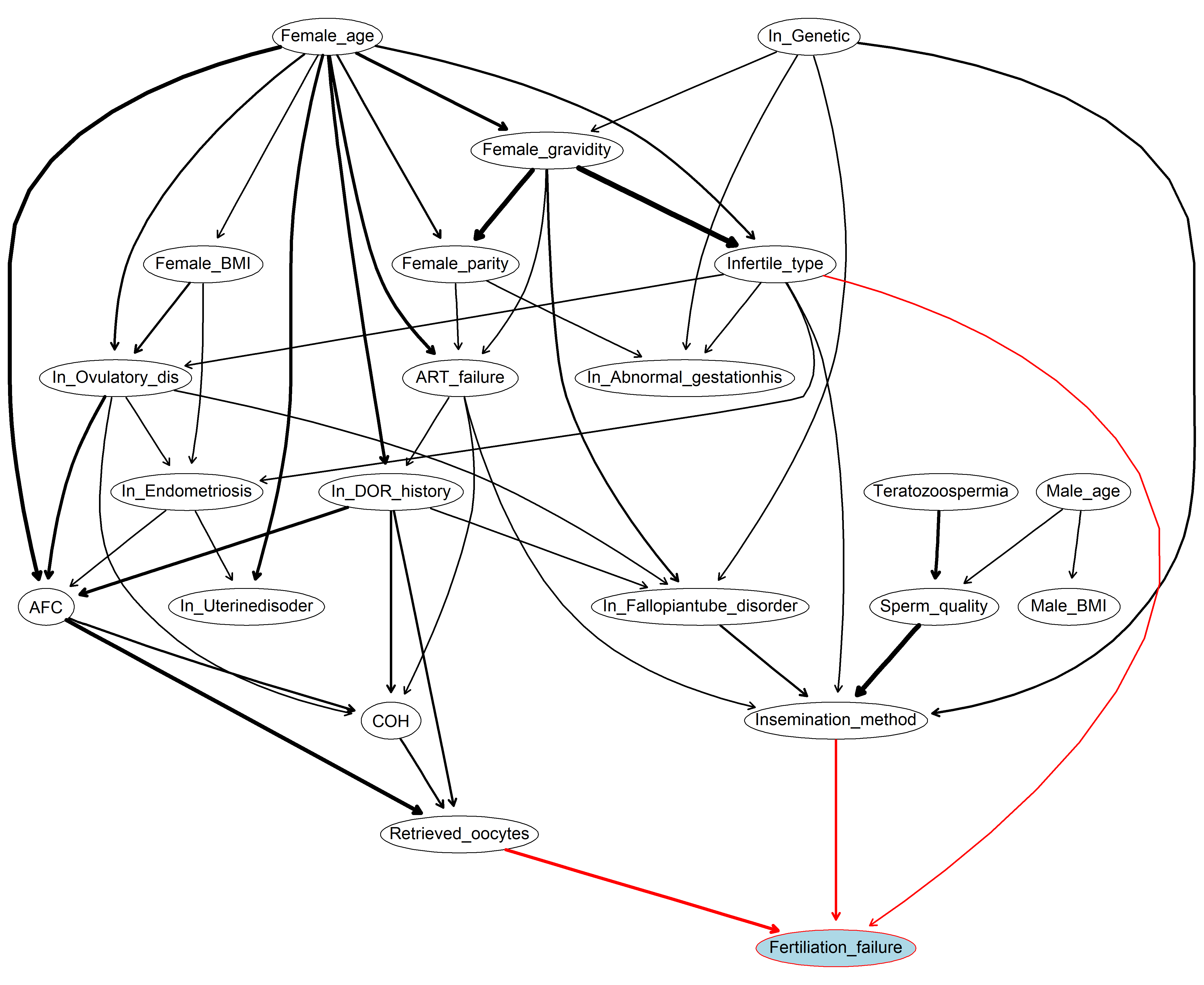


# Figure S2 Tenfold ten-cross validation


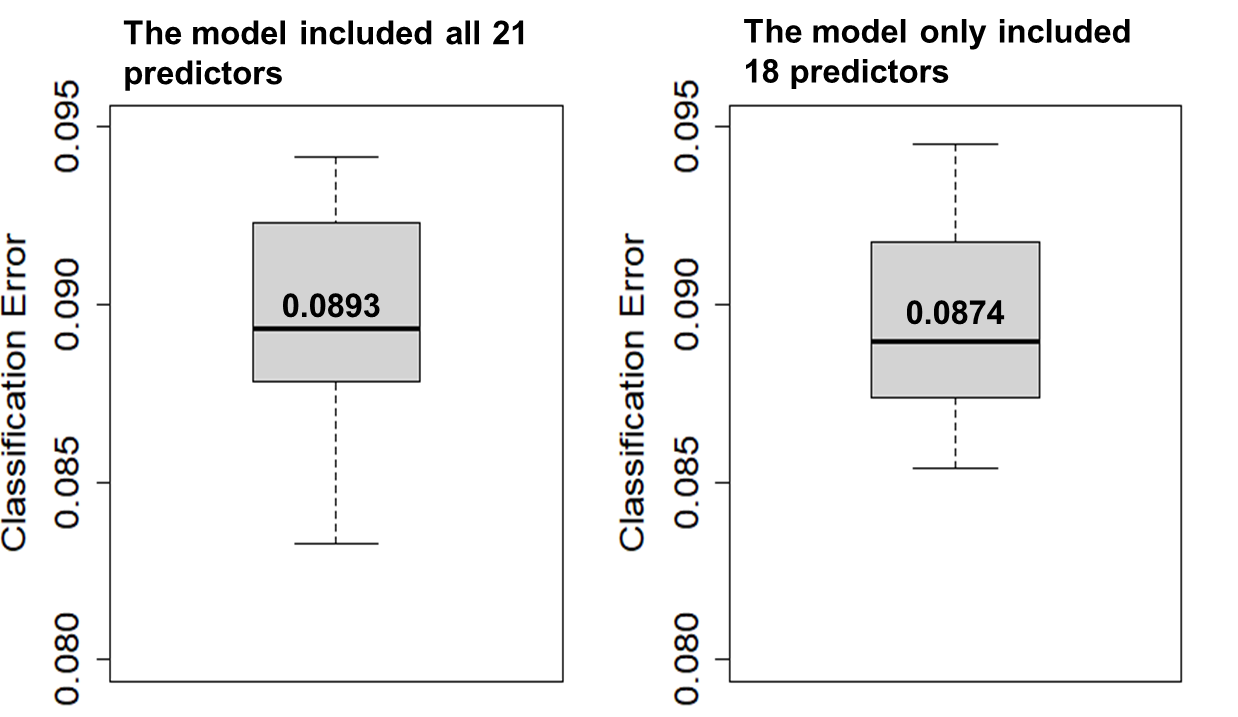

Supplement: Supplementary file 1 — Additional file 1: Table S1. The information of involved variables. Table S2. Predictor selection. Table S3. Comparison between included and excluded data. Figure S1. Bayesian Network model based on all predictors. Figure S2. Tenfold ten-cross validation. [file 12958_2023_1065_MOESM1_ESM.docx]
